# The Panitumumab EGFR Complex Reveals a Binding Mechanism That Overcomes Cetuximab Induced Resistance

**DOI:** 10.1371/journal.pone.0163366

**Published:** 2016-09-22

**Authors:** E. Allen Sickmier, Robert J. M. Kurzeja, Klaus Michelsen, Mukta Vazir, Evelyn Yang, Andrew S. Tasker

**Affiliations:** 1 Depatment of Therapeutic Discovery, Amgen Inc., 360 Binney Street, Cambridge, MA 02142, United States of America; 2 Department of Metabolic Disorders, Amgen Inc., One Amgen Center Drive, Thousand Oaks, CA 91320, United States of America; 3 Department of Therapeutic Discovery, Amgen Inc., One Amgen Center Drive, Thousand Oaks, CA 91320, United States of America; Technische Universitat Dresden, GERMANY

## Abstract

Panitumumab and cetuximab target the epidermal growth factor receptor for the treatment of metastatic colorectal cancer. These therapies provide a significant survival benefit to patients with metastatic colorectal cancer with wild-type *RAS*. A single point mutation in the ectodomain of EGFR (S468R) confers acquired or secondary resistance in cetuximab treated patients, which is not observed in panitumumab-treated patients. Structural and biophysical studies presented here show this mutation directly blocks cetuximab binding to EGFR domain III and describes a unique mechanism by which panitumumab uses a central cavity to accommodate this mutation.

## Introduction

The epidermal growth factor receptor (EGFR) regulates critical cellular processes such as proliferation, differentiation, and apoptosis, and is a major target of monoclonal antibodies in the oncology setting [1]. The two FDA approved antibody therapies that target the ectodomain of EGFR are the mouse/human chimeric IgG1 cetuximab and the fully human IgG2 antibody panitumumab [2]. Both antibodies prolong survival in patients with metastatic colorectal cancer [3,4,5]. Primary and acquired resistance to these antibodies can occur through mutations in KRAS, BRAF and NRAS that lead to aberrant activation of mitogen-activated protein kinase signaling [6,7,8,9]. In addition to mutations in downstream signaling molecules, mutations in the ectodomain of the EGFR exhibit resistance to antibody therapies [10]. An established mutation in the EGFR ectodomain S492R (S468R in the mature protein sequence and the numbering used here to be consistent with previous published structures) imparts resistance to cetuximab, yet strikingly remains responsive to panitumumab [10]. DNA sequencing showed this mutation results from a C to A substitution at nucleotide 1,476 (C1476A) and this mutation causes substitution of serine to arginine at amino acid 468 (S468R). Epitope mapping has shown that both cetuximab and panitumumab share the same epitope and both are considered equivalent treatments [10,11,12]. However, additional acquired mutations in the ectodomain of EGFR have also shown different resistance profiles to panitumumab and cetuximab [13, 14]. Given this difference in treatment response, we sought to understand panitumumab’s ability to target this mutation at the molecular level.

## Materials and Methods

### Protein Expression

Human EGFR domain III constructs EGFRD3 ND2 and EGFRD3 ND2(S468R) were expressed in baculovirus-infected Sf9 cells (Expression Systems). A 6X Histidine-cleavable tag was introduced to the C-terminus of the pFastBac vector (Invitrogen) containing EGFR with the native signal sequence in the 311–514 amino acid backbone with the following glycosylation mutations N328D and N420D. A second construct that contained the S468R mutation was created with the glycosylation mutations, N328D and N420D. Virus was prepared using the Bac-to Bac baculovirus expression system (GibcoBRL) and viral stocks were made and amplified in Sf9 cells. Expression was carried out in a Wave Cell Culture bag (GE Healthcare) at 1% virus inoculation at 27°C. Cells were harvested by centrifugation and the conditioned media was collected for purification of the secreted EGFRD3 ND2 or EGFRD3 ND2(S468R).

### Purification of EGFRD3 ND2 and EGFRD3 ND2(S468R)

25 L of conditioned media from Sf9 cells was filtered (0.45μm) and subjected to UF/DF using a 10K spiral cartridge (Amicon). The media was concentrated to 0.5L and exchanged into 20 mM Tris (pH 8.0), 100 mM NaCl and 20 mM imidazole prior to batch binding with 15 mL of Ni-NTA resin (Qiagen) for 1.5 h at 4°C. The resin was eluted with the same buffer at 250 mM imidazole. Pooled fractions were concentrated to less than 10 mL and injected onto a Superdex 200 XK26/60 (GE Healthcare) column equilibrated with; 25 mM HEPES (pH 8.0) and 100 mM NaCl. The SEC peak corresponding to monomeric EGFR was pooled and protein concentration was determined according to the molar extinction coefficient at 280 nm (35 mg recovery). Edman sequencing confirmed the processing of the N-terminal 24 aa leader sequence and LC-MS demonstrated partial glycosylation. The protein was frozen in liquid nitrogen and placed at -80°C for extended storage. Both EGFRD3 ND2 and EGFRD3 ND2(S468R) were purified by the same method.

### Purification of the Fab Complex

The Fab fragment of panitumumab was prepared by papain cleavage using immobilized papain (Themo Scientific). Papain was removed by centrifugation and the supernatant was loaded onto a Superdex 200 column to separate Fab from undigested material in a SEC buffer of 100 mM NaCl and 10 mM Tris-HCl 7.5. The panitumumab Fab was complexed with EGFRD3 ND2 or EGFRD3 ND2(S468R) in a 1:1.5 molar ratio of Fab:EGFR and run over SEC to purify the complex. The purified complex was concentrated to 15 mg/mL in 100 mM NaCl and 10 mM Tris-HCl 7.5. The His-tag was not removed from EGFRD3 ND2 and EGFRD3 ND2(S468R) prior to crystallization.

### Crystallization, Data Collection and Structure Determination

The panitumumab fab complexed with EGFRD3 ND2 was crystallized by the hanging drop vapor diffusion method. Drops contained equal parts EGFRD3 ND2 (15 mg/ml in 100 mM NaCl and Tris-HCl 7.5) and a precipitant solution containing 2.0 M AmSO4, 100 mM MES 6.5 and 5% peg 400 were equilibrated over a reservoir of precipitant solution at 18°C. Crystals were flash frozen in a precipitant solution supplemented with 10% glycerol and 10% ethylene glycol. X-ray diffraction data were collected using 1.0 Å wavelength at Advanced Light Source Beamline 5.0.2 (Lawrence Berkeley National Laboratory, Berkeley, CA) at 100 K. Data was processed and scaled using HKL2000 [15]. The crystals belong to the space group P2_1_2_1_2_1_ with approximate unit cell dimensions of *a* = 65 Å *b* = 113 Å, *c* = 231 Å. Molecular replacement was performed with Phaser [16], using an unpublished Fab structure of panitumumab and a truncated domain III of sEGFR (PDB:1YY9) as search models. The structure was refined using PHENIX [17], and the model was built with Coot [18]. Structure quality was assessed by Coot and PHENIX [17] validation tools. The EGFRD3 ND2 model contains 96% of residues in favored, 3.8% in allowed and 0% in outliers. The panitumumab Fab complexed with EGFRD3 ND2 (S468R) was crystallized exactly as described above for EGFRD3 ND2. The EGFRD3 ND2 (S468R) model contains 97% of residues in the favored, 3% in allowed and 0% in outliers. Ramachandran space was determined by PHENIX. Data collection and refinement statistics are summarized in Table 1.

**Table 1:** Data collection and refinement statistics.

	EGFRD3 ND2	EGFRD3 ND2(S468R)
**Data collection**		
Space group	P 21 21 21	P 21 21 21
Cell dimensions		
*a*, *b*, *c* (Å)	65.27, 113.18, 232.44	65.07, 113.10, 231.40
α, β, γ (°)	90, 90, 90	90, 90, 90
Resolution (Å)*	30–2.8 (2.9–2.8)	30–2.5 (2.59–2.5)
*R*_sym_ or *R*_merge_*	0.09 (0.52)	0.046 (0.45)
*I* / σI*	13.4 (2.0)	14.2(2.2)
Completeness (%)*	99.6 (98.6)	97.6 (92.0)
Redundancy*	6.0 (5.2)	4.1(3.8)
**Refinement**		
Resolution (Å)	30–2.8	30–2.5
No. reflections*	39087 (1930)	55059 (3812)
*R*_work_ / *R*_free_	0.223/0.266	0.226/0.249
No. atoms	9504	9644
Protein	9305	9361
Ligand/ion	82	144
Water	117	139
*B*-factors	54.62	55.59
Protein	54.76	55.69
Ligand/ion	70.32	69.19
Water	32.08	35.05
R.m.s. deviations		
Bond lengths (Å)	0.002	0.002
Bond angles (°)	0.49	0.50

*Values in parentheses are for highest-resolution shell.

### Surface Plasmon Resonance

SPR experiments were conducted on a Biacore T200 instrument (GE Healthcare). Panitumumab and cetuximab were immobilized onto a carboxymethyl dextran (CM5) biosensor chip using amine coupling chemistry at 25°C. Running buffer was compromised of 20 mM sodium phosphate pH 7.5, 150 mM sodium chloride and 0.05% (v/v) Tween20. Surface was activated for 7 minutes with a mixture of EDC and NHS (according to manufacturer’s instructions), 5 μg/ml of antibody diluted in 10 mM sodium acetate pH 5.5 was injected to a density of approximately 300 Response Units (RU) and remaining unreacted, NHS-activated carboxy groups was blocked for 7 minutes with 1 M ethanolamine, pH 8.5. Binding analysis was performed at 37°C using single site kinetics method and five consecutive injections of increasing concentrations of EGFR WT and S468R: 1.23, 3.7, 11.1, 33.3 and 100 nM (in duplicate). Antibody-EGFR association was observed for 2 minutes and dissociation for 30 minutes at a flow rate of 90 μl/minute. Data was processed using Biacore Evaluation software (Version 2.0, GE Healthcare) and data fit to a 1:1 binding model to establish kinetic binding parameters and ultimately affinity constant. Measurements were performed at least twice to allow calculation of a standard deviation.

## Results and Discussion

Initial preparations of the recombinant domain III of the EGFR ectodomain exhibited a high degree of heterogeneous glycosylation by the insect cell host and failed to produce co-crystals with the Fab fragment of panitumumab. A series of deglycosylated mutants were designed by making different combinations of amino acid replacements at the putative glycosylation sites. Whereas the most dramatic substitutions of four asparagine residues to aspartate resulted in an insoluble protein, a double mutant (N328D and N420D) was identified that exhibited reduced glycosylation and increased solubility. This construct, as well as a version that contained the S468R variation, were used for subsequent structural and biophysical studies. They are referred to as EGFRD3 ND2 and EGFRD3 ND2(S468R), respectively.

**Fig 1:**
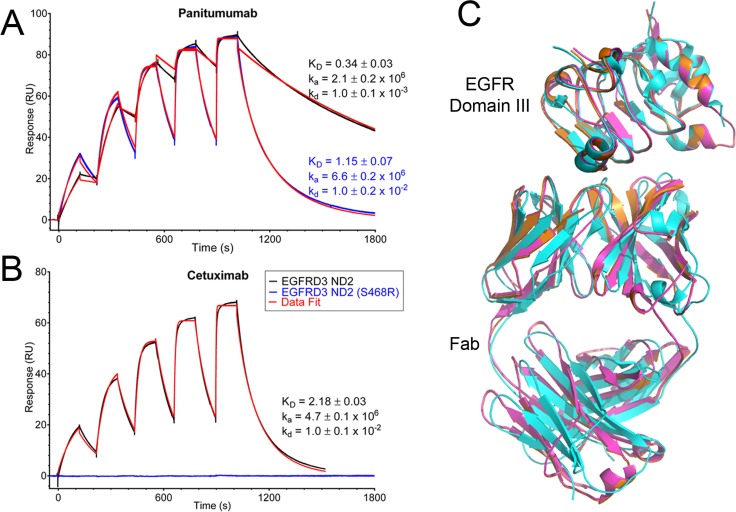
Comparison of panitumumab and cetuximab binding. (A) Surface plasmon resonance of EGFR binding to panitumumab and cetuximab. Increasing concentrations of EGFR construct (1.23, 3.7, 11.1, 33.3 and 100 nM) were injected over immobilized antibody. In black and blue are the raw binding traces of the two different EGFR constructs and in red is data fit to a 1:1 binding model. (B) No binding was observed for cetuximab and EGFRD3 ND2 (S468R), blue line. (C) Overlay of panitumumab and EGFRD3 ND2 is shown in orange, panitumumab and EGFRD3 ND2(S468R) in magenta and cetuximab and EGFR domain III in cyan. (PDB: 1YY9). Only EGFR domain III (not domains I, II, and IV) is shown in the cetuximab complex for clarity. Panitumumab and cetuximab recognize the same epitope.

Surface plasmon resonance (SPR) spectroscopy was used to assess the binding of panitumumab and cetuximab to the two different EGFR constructs. Panitumumab bound EGFRD3 ND2 with a K_D_ value of 0.34 ± 0.03 nM, while it bound the S468R mutant with a 3-fold less potent K_D_ of 1.15 ± 0.07 nM (Fig 1A). Cetuximab bound EGFRD3 ND2 with a significantly weaker K_D_ value (6-fold) of 2.18 ± 0.03 nM compared to panitumumab (Fig 1B) and the affinity was consistent with published values [19]. This lower affinity does not translate to a reduced *in vivo* efficacy as both medications are equivalent treatments. However, cetuximab showed no measureable binding to the S468R mutant supportive of the finding that the S468R mutation exhibits clinical resistance to cetuximab [10,20]. Notably, the affinity of panitumumab for the EGFR S468R mutation was still 2-fold better than the affinity of cetuximab for EGFRD3 ND2 which explains its therapeutic effect *in vivo* [10].

We determined the crystal structure of the panitumumab Fab in complex with EGFRD3 ND2 and EGFRD3 ND2(S468R). The binding site of panitumumab partially overlaps the EGF binding site and prevents the EGFR ectodomain from forming a dimerization-competent conformation [21,19]. The overall binding of the panitumumab Fab to EGFR domain III is very similar to the binding of cetuximab[19]. Cetuximab was previously shown to bind exclusively to domain III and the epitope for panitumumab is also confined to domain III (Fig 1C).

**Fig 2:**
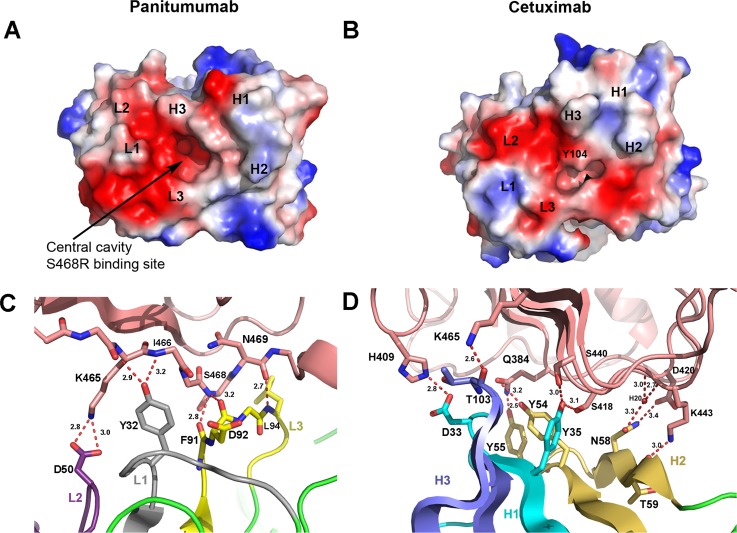
Paratope surfaces and CDR interactions. (A) Electrostatic surface of the CDR region of panitumumab. Large negatively charged cavity in center of CDR regions is clearly visible. (B) Electrostatic surface of CDR region for cetuximab reveals that Y104 fills the central cavity. (C) Light chain CDR interactions with the final β-strand of EGFR domain III. EGFR in tan with L1, L2 and L3 colored gray, purple, and yellow, respectively. (D) Heavy chain CDR interactions with the β-sheet surface of EGFR domain III that binds EGF. EGFR in tan with H1, H2, and H3 in cyan, gold and blue, respectively.

The solvent accessible surface area on domain III buried by the panitumumab Fab is 840 Å^2^ and the total solvent accessible surface buried at the panitumumab:domain III interface is 1664 Å^2^. This is slightly less than the buried surface area of cetuximab:domain III interface which is 1738 Å^2^ as calculated by PISA [22]. The panitumumab heavy chain complementarity determining regions (CDRs) and light chain CDRs all contact domain III of the EGFR. The CDRs except H3 pack against their corresponding Fab chain. This CDR arrangement, along with the configuration of the side chains in the beta sheet portion of the CDRs creates a central cavity located between the heavy and light chain (Fig 2A). Interestingly, this cavity is not filled by EGFR when bound to EGFRD3 ND2 and the conformation of the Fab is not altered significantly upon binding to the EGFR (unpublished panitumumab Fab structure).

The light chain CDRs make fewer overall interactions with domain III than the heavy chain CDRs and this is in agreement with what is typically observed for antibodies [23,24]. Y32 of L1 makes a bifurcated hydrogen bond between the carbonyl of K465 and the NH of I466 of domain III (Fig 2C). The L2 CDR makes only a single interaction through a solvent exposed salt bridge between D50 and K465 of domain III. This is consistent with the general observation that L2 often makes the smallest binding contribution and that CDRs H2, H3, and L3 commonly make the largest contributions to antigen binding [24]. The L3 CDR lines up adjacent to the final β-strand of domain III and makes main-chain to main-chain interactions between the carbonyl oxygens of F91 and D92 and the NH of S468 of domain III (Fig 2C). An additional L3 interaction is made by the NH of L94 and the carbonyl oxygen of N469 of domain III.

**Fig 3:**
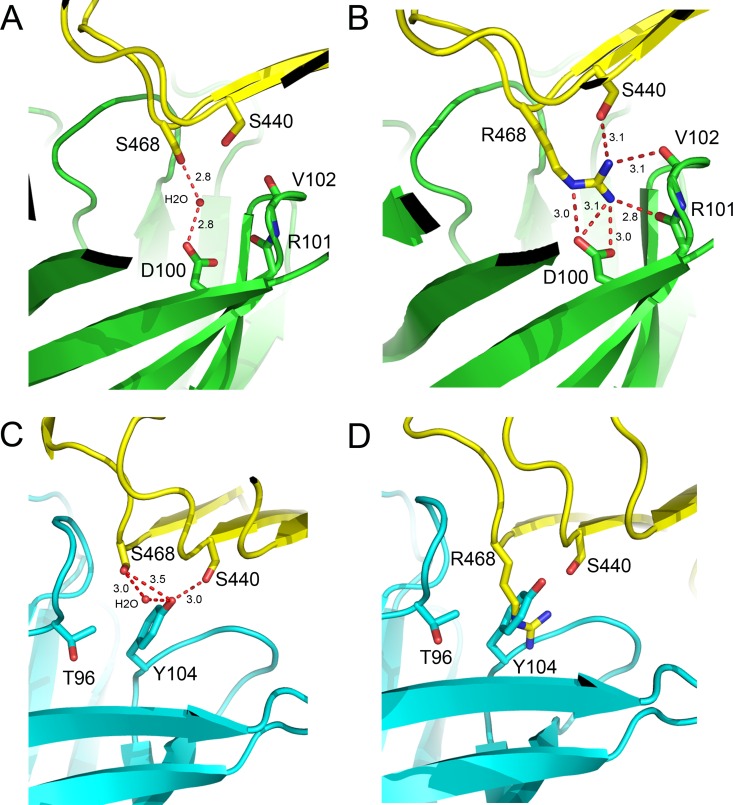
Comparison of residue 468 binding sites. (A) Binding site of panitumumab with EGFRD3 ND2. The S468 interaction with D100 is mediated through a water molecule. (B) Binding site of panitumumab with EGFRD3 ND2(S468R). R468 makes hydrogen bond interactions with D100, S440 and the main chain carbonyls of R101 and V102. (C) Binding site of cetuximab with S468 (PDB: 1YY9). Y104 packs against the S468 position and would not tolerate a large side chain in that position. (D) Model of EGFR S468R bound to cetuximab. The EGFR S468R mutation would severely clash with Y104. Model created by overlaying EGFRD3 ND2(S468R) onto EGFR domain III in the cetuximab structure without further refinement.

The heavy chain CDRs account for the majority of the buried surface area (63%) with most of the specific hydrogen bond interactions at the panitumumab:domain III interface being made by CDRs H2 and H3 (Fig 2D). Most notably three tyrosine residues interact with the same β-stand surface of EGFR domain III that interacts with EGF. Y35 of H2 hydrogen bonds with S440 and S418 of domain III and H3 residues Y54 and Y55 which hydrogen bonds to N384 of domain III. Additional H2 hydrogen bonds include the carbonyl of T59 to K443 of domain III, which is highly solvent exposed, and H2 residue N58 to D420 and a water molecule which in turn makes water mediated interactions with carbonyl and the main-chain NH of D420 and K443, respectfully. H3 makes an additional hydrogen bond between the carbonyl of T103 and K465 of domain III which is also highly solvent exposed. A single water mediated interaction by the H3 CDR is visible in the central cavity of the EGFRD3 ND2 Fab complex (Fig 3A). The water mediates an interaction between Ser468 on the EGFR and Asp100 in the H3 CDR. In the EGFRD3 ND2(S468R) Fab complex, the guanidinium group of Arg468 sits approximately where the water was positioned in the EGFRD3 ND2 Fab complex (Fig 3B). The Nɛ of Arg468 mimics the water interaction by forming a hydrogen bond to the OD2 of Asp100. The Arg468 NH2 sits within about 3.0 Å of OD1 and OD2 of Asp100 and the carbonyl of Arg101 in H3. The remaining NH1 is positioned 3.1 Å from Ser440 of the EGFR and the carbonyl of Val102 in H3. This mutation is accommodated in the cavity with only a minor 0.5 Å movement of H3 which makes most of the electrostatic interactions with Arg468. The pocket has an overall negative charge that may tolerate hydrophobic or positively charged mutations more easily. However, as seen with the Ser468 in the EGFRD3 ND2, water mediated interactions can also provide charge tolerance, particularly for smaller side chains.

The previously determined cetuximab EGFR structure showed that cetuximab binds to the same epitope as panitumumab with a very slight counterclockwise rotation relative to the EGFR when viewed down the CDRs [19]. The Fab of cetuximab lacks the large central cavity seen in panitumumab and has only a small pocket formed between Tyr104 in H3 and the L3 loop which is also present in panitumumab as part of the larger central cavity (Fig 2A). The equivalent panitumumab cavity is filled primarily with Tyr104 from H3 in cetuximab which makes hydrogen bond contacts with Ser404 and Ser468 of EGFR (Fig 3C). Tyr104 would sterically block the binding of Arg468 in the S468R mutant (Fig 3D). The equivalent residue in panitumumab is Gly104 and the lack of a side chain creates this central cavity. The presence and position of Tyr104 explains the large difference in binding between panitumumab and cetuximab to this mutation.

**Fig 4:**
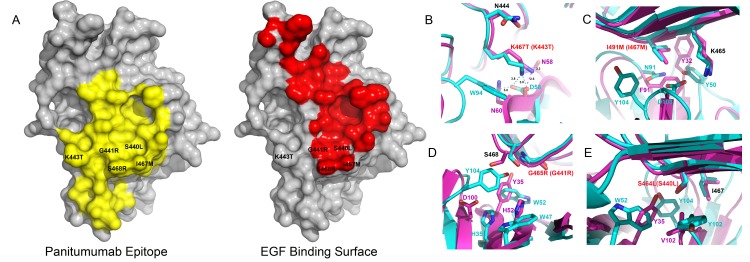
EGFR domain III mutants. (A) Panitumumab epitope on EGFR domain III of EGFR is shown in yellow. EGF binding surface on EGFR domain III is shown in red (PDB:1IVO). Mutations are shown on surface relative to panitumumab epitope and EGF binding interface. EGF and panitumumab have significant overlap in the domain III binding surfaces. S468R as well as many additional mutations are located on the perimeter of the EGF binding site but are centrally located in the panitumumab epitope. (B-E) Panitumumab is shown in magenta and cetuximab is shown in cyan. Red lettering represents mutation and the number in parenthesis represents numbering based on mature protein sequence used in the crystal structure. The remaining residues are numbered to be consistent with crystal structure numbering. (B)The K467T mutation will result in a clash with W94 in cetuximab but rotamer conformations exist that would minimize the steric clash in panitumumab, as a result cetuximab is sensitive to this mutation but panitumumab remains active. [13]. (C) I491M would result in steric clashes with D103 and Y102 in cetuximab and F91 and the main chain of T103 in panitumumab. (D) G465R would result in steric clashes with W52 and His35 in cetuximab and H52 and Y35 in panitumumab. (E) S464L is in a tightly packed region and would clash with Y102 and Y104 in cetuximab and V102 and Y35 in panitumumab.

Analysis of on-study plasma samples from the phase III ASPECCT trial has revealed that the S468R EGFR mutation emerges upon progression of treatment with cetuximab monotherapy [25,26]. After treatment with cetuximab 16% of patients developed the S468R mutation compared to 1% for the panitumumab arm. It is likely that only panitumumab is effective against the S468R mutation and therefore it does not significantly propagate this mutation in treated patients. This suggests that follow-up treatment of panitumumab in cetuximab treated patients may be warranted. Since both cetuximab and panitumumab have negatively charged surfaces and cetuximab lacks a central cavity it is harder to predict which single point mutations could allow binding of cetuximab but prevent panitumumab binding while maintaining a functional EGFR that can bind to EGF. The epitope of panitumumab and cetuximab partially overlaps with the EGF binding site (Fig 4A) and mutations would need to inhibit cetuximab or panitumumab but not block EGF binding in order to maintain a functional receptor. The S468R mutation is on the perimeter of the EGF binding site and blocks binding of cetuximab but not panitumumab despite being well inside panitumumab’s epitope.

Additional aquired muations that fall into the epitope binding site for cetuximab and panitumumab include K467T, G465R, S464L, and I491M (K443T, G441R, S440L and I467M in the mature protein sequence) [13, 14]. Only K467T shows resitance to cetuximab but remained sensitive to panitumumab. This mutation would disrupt a solvent exposed salt bridge between K467 of domain III and Asp56 in cetuximab and produce a steric clash with W94 in cetuximab (Fig 4B). Panitumumab lacks a large side chain and rotamer conformations for theronine exist that would minimize a steric clash and allow panitumumab to bind effectively. The remaining mutations all show clashes with neighboring residues (Fig 4C–4E) when bound to panitumumab or cetuximab. None of these mutations can access the central cavity and as a result they have similar negative effects on the binding for both cetuximab and panitumumab. An additonal mutation R451C is outside of the binding epitope of cetuximab and panitumumab and reduces the affinity of both panitumuab and cetuximab equivalently. This is likely due to the introduction of a cysteine residue in close proximity to the cysteine-rich extracellular domains which may alter disulfide binding patterns. Certainly, single point mutations that disrupt panitumumab but not cetuximab binding are possible but the large central cavity in panitumumab provides a novel avenue to accommodate mutations in this region that are outside of the EGF binding site.
